# In Vitro Assessment of *Boswellia serrata* Incorporated N95 Mask Layers ‐Specific Efficacy Comparison.

**DOI:** 10.1002/gch2.202500328

**Published:** 2025-11-19

**Authors:** Aiswarya K. Raj, Raichal B. George, Geetha B. Kumar, Jayalekshmi Haripriyan, Kamalam S. Rajni

**Affiliations:** ^1^ Department of Chemistry Amrita School of Physical Sciences Coimbatore Amrita Vishwa Vidyapeetham India; ^2^ AMRITA Lab Amrita School of Engineering Coimbatore Amrita Vishwa Vidyapeetham India; ^3^ Amrita School of Biotechnology Amrita Vishwa Vidyapeetham Amritapuri Kollam Kerala 690525 India; ^4^ Department of Physics Amrita School of Physical Sciences Coimbatore Amrita Vishwa Vidyapeetham India

**Keywords:** airborne diseases, antimicrobial, *Boswellia serrata*, N95 mask layer

## Abstract

This research focuses on designing a novel, five‐layered N95 mask fabric that integrates the natural antimicrobial properties of *Boswellia serrata*, thereby unlocking a new dimension in respiratory protection. Specifically, the second and third layers of the mask fabric were coated with a chloroform extract of *Boswellia serrata* to impart layer‐specific functionality. The functionalized mask fabrics underwent rigorous analysis, including Scanning Electron Microscopy (SEM), Energy Dispersive X‐ray (EDX) Spectroscopy, Fourier Transform Infrared Spectroscopy (FTIR), and wettability measurements, confirming the successful incorporation of the extract. The contact killing assay demonstrated a highly effective dual‐action defense system. The extract‐coated second layer exhibited a rapid, but transient, antimicrobial effect, showing excellent inhibition within one hour (92% against *S. aureus*, 86% against *E. aerogenes*), though this effect diminished significantly by eight hours. In contrast, the third layer provided a prolonged and sustained antimicrobial effect, maintaining high inhibition even after eight hours (100% against *C. albicans* and *K. pneumoniae*, and 90% against *E. aerogenes*). Maximum killing efficiency was observed at four hours for both layers. This innovative application of layer‐specific engineering offers enhanced and prolonged protection against airborne pathogens, marking a significant leap in mask technology.

## Graphical abstract

This study reveals the potential effect of Boswwllia serrata extract on various layers of mask fabrics. The paper provides a comprehensive detailing of methods and characterization of fabrics of mask layers coated with extract. This paper gives the efficacy comparison of different two layers of mask fabrics showing rapid antimicrobial activity of the second layer in comparison with the prolonged and sustained activity of the third layer.

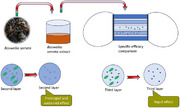

## 1 Introduction

During the recent global crisis of the Covid pandemic, facemasks played an important role in preventing transmission to a great extent. These facemasks are of various categories has different filtration capabilities such as cloth facemasks, surgical facemasks and respirators. The cloth facemask filters the dust particles, viruses and bacteria, but the filtration efficiency is very low.^[^ ^1^ ^]^ While the surgical facemask has a filtration efficiency of 60%–80% to filter viruses, bacteria and dust, and it is disposable.^[^ ^2^ ^]^ The N95 facemask, which belongs to the category of respirators, has 95% filtration efficiency for filtering various pathogens.^[^ ^3^ ^]^

Earlier studies have attempted to improve the effectiveness of facemasks by employing several amendments, including metals and salts, natural products, photosensitizers and graphene. Metal salts are used to fabricate functionalized facemasks by coating with starch‐capped AgNPs,^[^ ^4^ ^]^ copper oxide loaded polyacrylonitrile nanofibers,^[^ ^5^ ^]^ zinc (II) ions.^[^ ^6^ ^]^ Photosensitizers such as photocatalytic titanium dioxide filters^[^ ^7^ ^]^ photoactive conjugated polymers and oligomers,^[^ ^8^ ^]^ as well as the incorporation of graphene^[^ ^9^, ^10^ ^]^ have also been employed to modify the facemask. Individuals those who are prone to respiratory illness can easily attack by airborne pathogens and thereby increases their life threatens. Air‐borne diseases can be caused by various pathogens such as viruses^[^ ^11^, ^12^ ^]^ bacteria^[^ ^13^, ^14^, ^15^ ^]^ and fungi.^[^ ^16^, ^17^, ^18^ ^]^ So, use of functionalized facemask is extremely crucial for achieving significant protection against these infectious agents.

Despite these advancements, the efficacy of the filter layers of mask can be further enhanced by coating with potential natural products. However, the facemasks infused with plant extract and its enhanced efficacy has received limited attention. A deeper understanding of different functionalization of face mask by incorporating various bioactive molecules is essential for improving the impact on their antimicrobial properties. *Boswellia serrata*, commonly known as frankincense, has long been recognized for its antimicrobial properties^[^ ^19^ ^]^ and antiviral^[^ ^20^ ^]^ properties, the main constituent is boswellic acid, primarily attributed for various activities.^[^ ^21^ ^]^

In this study, we introduce a novel approach by modifying the filter layer of N95 masks by incorporating *Boswellia serrata* resin extract (BSE). This approach appreciably enhanced the ability of N95 masks to filter bacteria and fungi, paving the way for the development of more efficient and bioactive filter layer for next‐generation facemasks.

## 2 Experimental Section

### 2.1 Materials

*Boswellia serrata* gum resin was obtained from Idukki district, Kerala, India. Chloroform was purchased from SRL Chemicals. We purchased BIS approved FFP2 N95 face mask from TATA 1 mg brand.

### 2.2 Preparation of Extract

About 200 g of *Boswellia serrata* resin was powdered using mortar and pestle, and extracted using a conventional Soxhlet apparatus. About 50 g of the powdered resin was extracted using 250 mL of chloroform maintained at 55 °C for 12 h. The extracted resin was concentrated under reduced pressure.

### 2.3 Preparation of Coating Solution and Coating on N95 Mask Fabric

**Figure 1:**
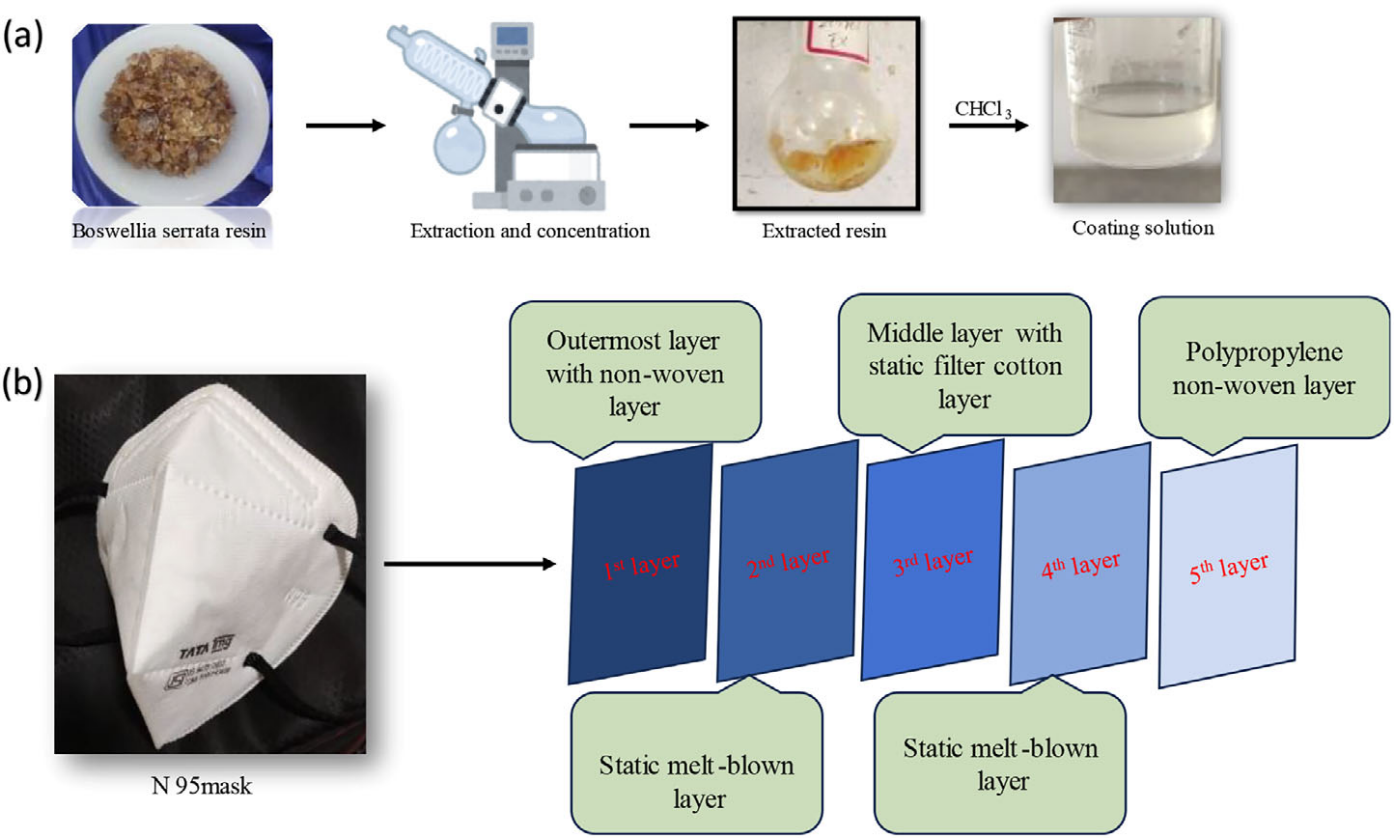
a) Preparation of coating solution and b) Various layers of N95 mask.

About 300 mg of the extracted resin was dissolved in 50 mL of chloroform to prepare the coating solution (**Figure** **1**a). The N95 masks purchased have five layers. The innermost layer or fifth layer is made up of polypropylene non‐woven fabric for filtering dust particles, and the second and fourth layers are made up of static melt‐blown fabric for filtering particles smaller than 0.3 microns, while the third layer was made of static cotton filter for filtering larger particles and the first layer or the outermost layer is comprised of non‐woven filter layer (Figure 1b). The focus was to modify the second and third layers of the N95 mask fabric by adsorbing the of BSE onto the surface and comparing their efficacy of the layers with respect to their antimicrobial activity. The second and third layer fabrics of the N95 mask were cut into small pieces of 3 cm x 1 cm, washed with ethanol and dried for 20 min at 80 °C using a hot air oven. Subsequently, the washed third (cotton) layer and the second (melt‐blown polypropylene) layer samples were dipped several times in the coating solution for 10 min. The flannels were then dried using a hot air oven for 2 h at 120 °C, following which the modified second and third layers were subjected to further analysis.

### 2.4 Characterization

#### 2.4.1 Field Emission Scanning Electron Microscopy: Energy Dispersive X‐Ray Spectroscopy (FESEM‐EDX) Analysis

SEM‐EDS analysis (Zeiss Gemini 300) was performed to observe the morphology of the second and third layer fabrics of the N95 mask that was coated with resin extract and compared with the uncoated fabric as a control.

#### 2.4.2 Fourier Transform Infrared Spectroscopy

Fourier transform infrared (FTIR) spectroscopy of samples coated with resin extract was carried out using the Bruker FTIR spectrometer instrument (Bruker Vertex 70, Germany) at a spectral range of 400–4000 cm^−1^ using ATR mode, with the uncoated fabric as the control.

#### 2.4.3 Contact Angle

The interaction of water and the surface of the modified samples coated with and without resin extract was analyzed by sessile drop technique, to measure the hydrophobic and hydrophilic nature of surfaces based on contact angle (Holmarc contact angle instrument).

### 2.5 Antimicrobial Studies

#### 2.5.1 Test Microbial Strains

The antibacterial and antifungal efficacy of BSE of the coated and uncoated fabric layers of the mask was quantitatively analyzed using agar diffusion assay and contact killing assay, against the gram‐negative bacteria *E. aerogenes* ATCC 13 048 and *K. pneumoniae* ATCC 33 495, the gram‐positive bacterium *S. aureus* MTCC 96 and the fungus *C. albicans* MTCC 189. Mueller Hinton (MH) agar/ broth (Himedia, India) was used to grow *E. aerogenes*, *K. pneumoniae* and *S. aureus*, while *C. albicans* was grown on Yeast Extract Potato Dextrose (YEPD) agar/ broth (Himedia, India).

#### 2.5.2 Agar Diffusion Methods

The agar well diffusion method was used to assess the antimicrobial activity of BSE. Logarithmic phase culture (OD _600_ = 0.2) of the various microbes, grown in MH agar or YEPD agar, was evenly spread using a cotton swab, to obtain a lawn culture. Wells were punctured in the agar and 25 µl of BSE at a concentration of 500 µg was added to each of the wells. The plates were incubated at 37 °C for 24 h and the zone of inhibition was measured.

#### 2.5.3 Broth Dilution Method

The broth dilution method was used to assess the antimicrobial activity of the BSE. Overnight cultures of *S*. *aureus*, *K*. *pneumoniae*, *E*. *aerogenes* were diluted in MH broth and *C. albicans* was dilutes in PDB broth and OD was adjusted to 0.2 at 600 nm. The microbial suspension along with extract at a concentration of 100 µg mL^−1^ was added in wells that were then spread out onto agar plate followed by incubation for 24 h at 37 °C. aliquots from the wells were then spread out onto agar plates and incubated for overnight at 37 °C. All experiments were performed in triplicate.

#### 2.5.4 Contact‐Killing Assay

A single bacterial colony was inoculated in 5 mL of MH broth and incubated at 37 °C (150 rpm) overnight. For further contact killing assay, *S. aureus, K. pneumoniae*, *E. aerogenes* and a fungal pathogen, *C. albicans* culture was diluted to an OD of 0.1 at 600 nm. 500 µL of the culture was added onto the surface of pre‐sterilized mask materials (which included coated and uncoated material) and incubated at 37 °C. At distinct time points, viz., 1 and 8 h, the samples were vortexed with 1 mL of sterile PBS to harvest the microbes and the suspension was serially diluted and spread on MH and YEPD agar plates. The colony count was determined after overnight incubation at 37 °C.^[^ ^22^ ^]^

### 2.6 Bacterial Filtration Efficiency and Breathability

According to IS 16 288, the third layer of mask fabric with an area of 15 × 15 is conditioned in standard atmospheric temperature of 21 °C and a relative humidity of 85% for 4 h. The face side of the layer was tested against aureus (ATCC 6538) with a concentration of 5 × 10^5^ CFU mL^−1^ using a flow rate of 28.3 L min^−1^ having a mean particle size of 3.0 micron. The breathability of the third layer of mask material was analyzed using IS 16 289 : 2019 method with a tested area of 4.9 cm^2^ and with a flow rate of 8 L min^−1^.

## 3 Results and Discussion

### 3.1 The Physical Appearance of Fabrics Modified with Extract

**Figure 2:**
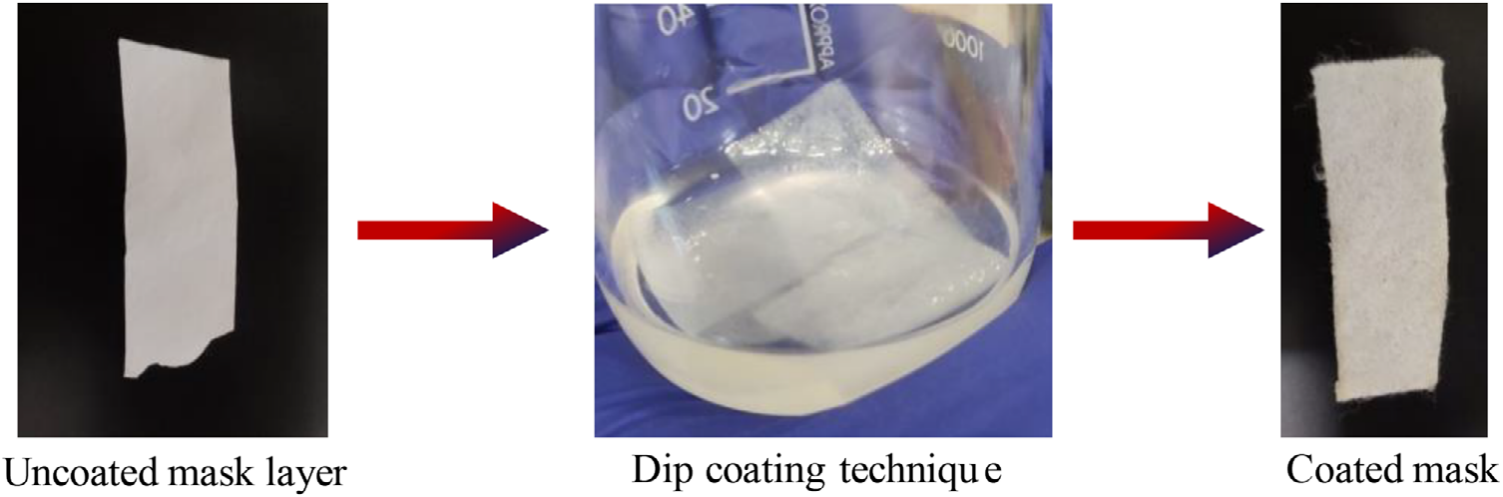
Physisorption of the mask layer by dip coating method.

The appearance of the polypropylene layer and the cotton layer modified using BSE transformed to a pale‐yellow color, due to the physisorption of the bioactive constituent present in the extract on the surface of the fabrics.^[^ ^23^ ^]^ The main bioactive constituent of the *Boswellia serrata* is boswellic acid and its derivative. The boswellic acid is hydrophobic and contains a carboxylic acid (‐COOH) and hydroxyl (‐OH) functional group. These moieties can interact with the hydroxyl (‐OH) groups present in the cotton fabric of the middle layer of the mask fabric, leading to the physical adsorption. The hydrophobic nature of the polypropylene (second) fabric of the mask as well as the boswellic acid, resulted in physical interaction driven by weak van der Waals forces. The extract is dissolved in a volatile solvent followed by the material is dipped in the solution and subsequently allowing the solvent to evaporate results a fine layer of extract was formed on the exterior surface of the polypropylene layer as well as the cotton layer. The yellowish coloration observed on the surfaces of the second and third layers were confirmed the successful adsorption of the resin extract (**Figure** **2**).

### 3.2 Field Emission Scanning Electron Microscopy (FESEM) analysis

**Figure 3:**
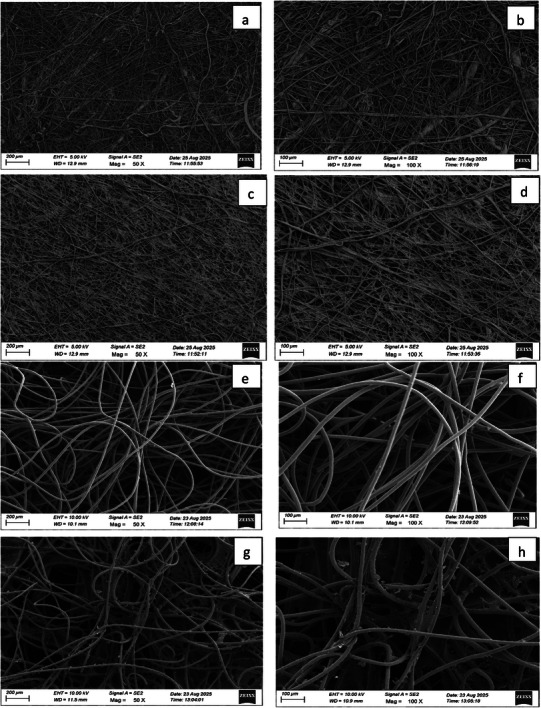
Scanning electron microscopy images of a,b) uncoated second layer, c,d) extract coated on the second layer, e,f) uncoated third layer, (g and h) extract coated on the third layer, at different magnifications.

The surface morphology was studied using scanning electron microscopy technique for the samples with and without coating of BSE. The morphology of the second layer made up of polypropylene and the third layer made up of cotton was examined at different magnifications found to be comprised of microtube‐like structures revealing distinct characteristics. The SEM images of the fabrics of mask layers coated with BSE reveals the presence of bioactive components on the surface of the microtubules across the fabric. These micrographs indicated that there are notable morphological changes in the second and third layers because of the adsorption of BSE on the surface of each layer,^[^ ^24^, ^25^ ^]^ as depicted in the SEM micrograph (**Figure** **3**). The Figure 3a,b represents SEM images of the uncoated second layer made up of polypropylene material at a scale of 200 and 100 µm with different image magnification of 50X and 100X respectively. The adsorption of the extract on the surface of the second layer can be confirmed from the Figure 3c,d, showing the dense microtubular structures in which the extract is absorbed and adsorbed on its surface. The presence of cavities in the images indicates the evaporation of the solvent by retaining the bioactive components in the extract on the surface of the material. Figure 3e,f represents SEM micrographs of the third layer of mask coated with cotton fabric at a scale of 200 µm and 100 µm with different image magnification of 50X and 100X respectively. From the Figure 3g,h, it is evident that the extract is coated uniformly on the third layer fabric of the mask by comparing with the uncoated layer.

### 3.3 Energy Dispersive X‐Ray (EDX) Spectroscopy

**Figure 4:**
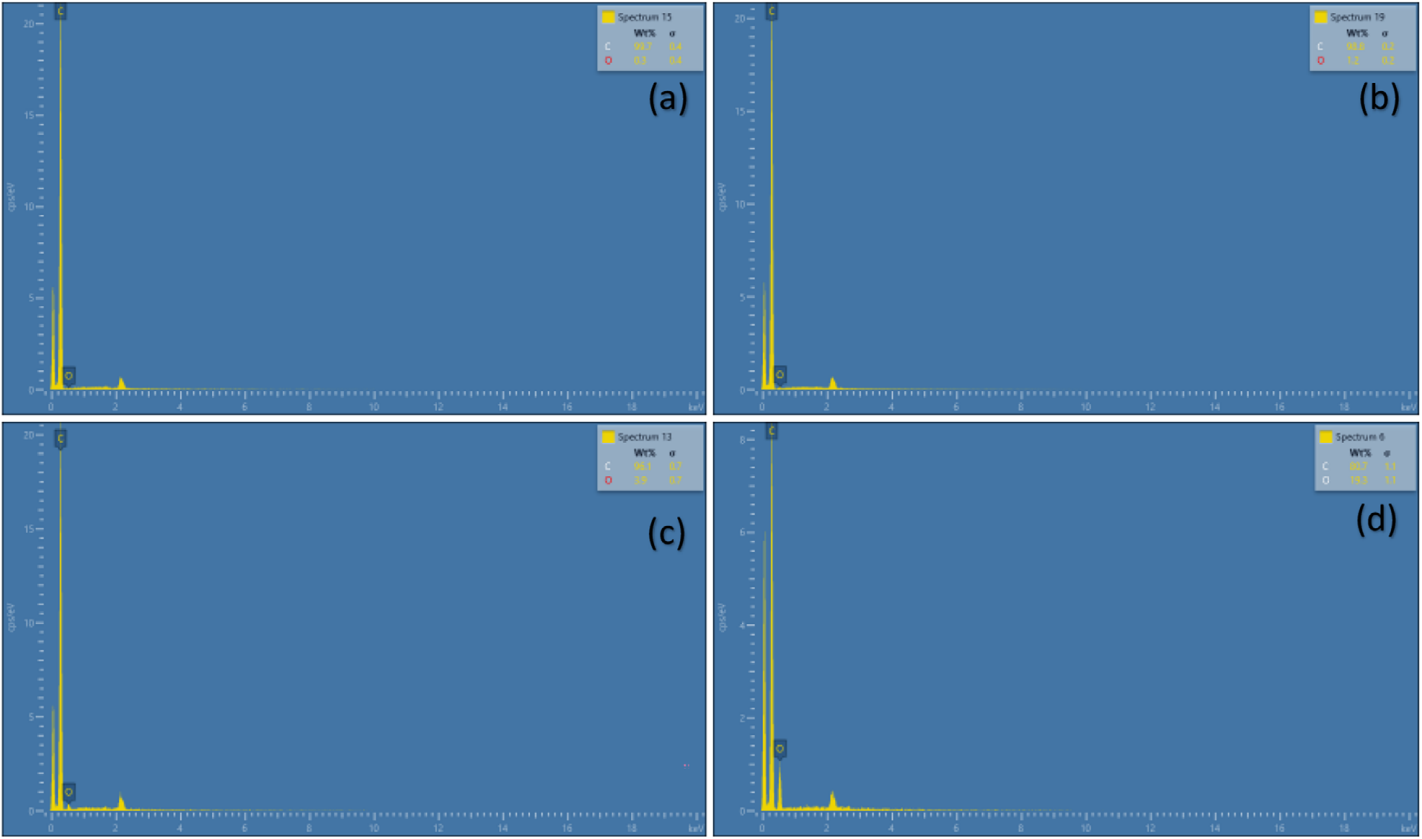
Energy dispersive pattern of a) uncoated second layer, b) extract coated second layer, c) uncoated third layer, and d) extract coated third layer.

The presence of carbon and oxygen on the uncoated layers and extract coated layers was confirmed by EDX analysis. **Figure** **4**a,c represents EDX pattern of the uncoated second and third layer of mask fabric is made up of polypropylene and cotton material respectively. The EDX pattern of the second and third layers of mask coated with BSE was represented by the Figure 4b,d respectively. The EDX analysis of further used for the validation of presence of residual solvent in the mask. The evaporation of the solvent used was confirmed by the absence of chlorine from the EDX pattern, which reveals there is no chloroform retains on the surface of the mask layers. The elemental composition of each element present in the samples were depicted in the **Table** **1**. It is found that the uncoated second layer has highest percentage of carbon and on coating with extract the percentage of carbon is decreased from 99.71% to 96.29%, while the percentage of oxygen is increased from 0.29% to 3.71%. The percentage of carbon on the uncoated and coated third layer of mask was decreased from 98.16% to 80.73% respectively, while the percentage of oxygen is increased 1.84% to 19.27%. By comparing the percentage composition of carbon and oxygen on the uncoated and coated layers of mask, it is found that the percentage of carbon is decreased while the percentage of oxygen is increased for the extract coated mask layers compared to uncoated mask layers. From the composition, it is evident that the extract is absorbed on the surface of the mask layers followed by increase in the percentage of oxygen. The weight percentage of carbon and oxygen is tabulated in Table 1. The elemental composition of carbon and oxygen in coated layers decreased due to the resin extract being trapped in the layer.^[^ ^26^ ^]^

**Table 1:** The elemental composition of elements on each layer.

Layers	Carbon [%]	Oxygen [%]
Second layer	99.71	0.29
Second layer with extract	96.29	3.71
Third layer	98.16	1.84
Third layer with extract	80.73	19.27

### 3.4 Fourier Transform Infrared Spectroscopy

**Figure 5:**
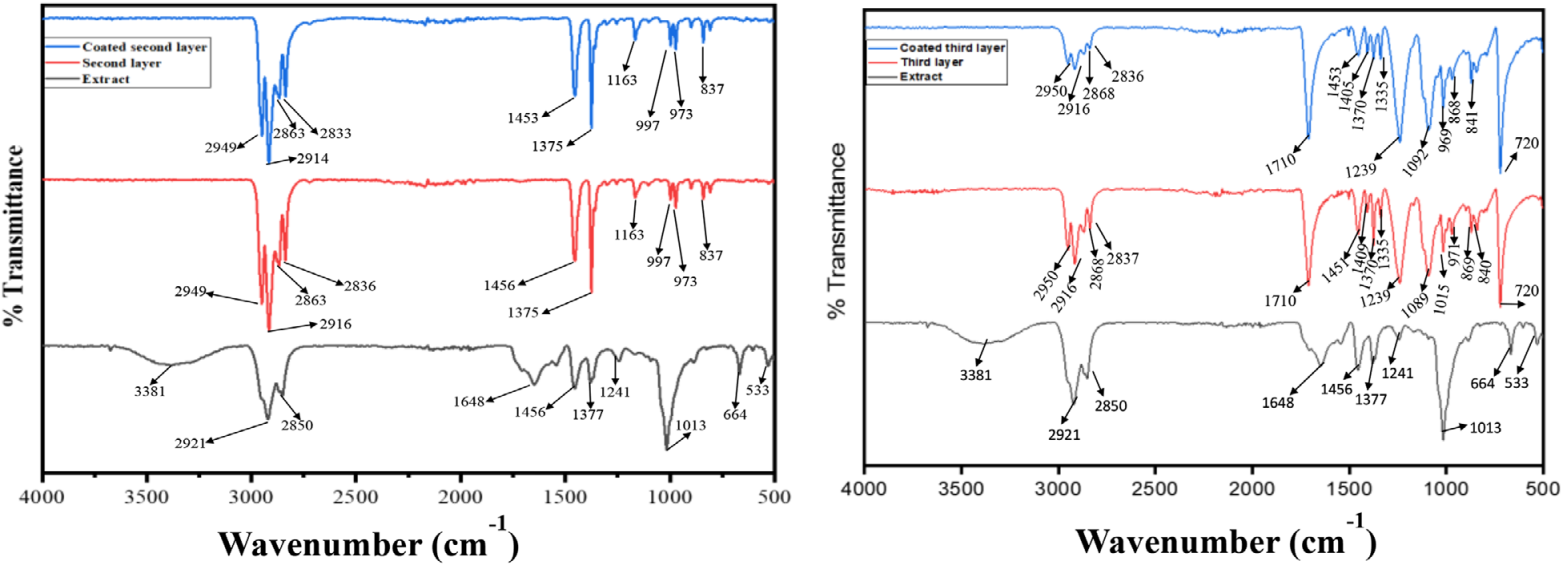
Represents the FTIR spectrum of a) the second layer of the coated and uncoated fabrics of mask, and b) the third layer of coated and uncoated fabrics of mask.

The second and third layer of uncoated mask and BSE coated mask layers were further validated by Fourier transform infrared (FTIR) analysis. The presence of various functional group in the BSE, uncoated and extract coated fabrics of mask is elucidated using FTIR spectroscopic characterization which is depicted in **Figure** **5**.

The spectra of the extract show a wide peak at 3381 cm^−1^, an intense pointed peak at 2921 and 2850 cm^−1^, which is due to the stretching of the hydroxyl group in the carboxylic acid.^[^ ^27^ ^]^ The broad vibration band at 1648 shows the presence of C=O hydrogen‐bonded stretching vibrations.^[^ ^28^ ^]^ The CH3 bending vibration is observed at 1456 and 1377 cm^−1^.^[^ ^29^ ^]^ The presence of CH_2_ twisting is observed by the peak at 1241 cm^−1^.^[^ ^30^ ^]^ The C‐C and C‐O bond shows stretching vibration which can be interpreted from the peak at 1013 cm^−1^.^[^ ^31^ ^]^

The uncoated and coated second layer shows characteristic bands at 2946 to 836 cm^−1^. The uncoated and coated layers have strong, intense sharp peaks at 2949, 2916, 2914, and 2863 cm^−1^ due to the asymmetric bending vibrations of C‐H bonds. The intense, sharp peak at 2836 cm^−1^ on the uncoated second layer and 2833 cm^−1^ on the coated second layer is due to the asymmetric and symmetric stretching vibrations of C‐H of CH_2_ and CH_3_ group.^[^ ^32^ ^]^ The asymmetric bending of the C─H bond of CH_3_ in the uncoated layer is observed as a strong peak at 1456 cm^−1^ and in the coated layer at 1453 cm^−1^. The strong, intense band at 1375 cm^−1^ can be attributed to the symmetric bending of the C─H bond of CH_3_ in both coated and uncoated second layers. The observed peak at 1163 cm^−1^ is due to the stretching of the C─C bond, wagging of the C─H bond and rocking of CH_3_ in the uncoated and coated second layer. The rocking vibration of CH_3_ in both the coated and uncoated second layer can be observed again at 997 cm^−1^. The less intense sharp peak at 973 cm^−1^ is observed due to the rocking vibration of the CH_3_ group and the stretching vibration of the C─C bond in the coated and uncoated second layer. The rocking vibration of the C─H bond can be observed as a less intense peak at 837 cm^−1^ in both the uncoated and the coated second layer.^[^ ^33^ ^]^ The second layer coated with Boswellia resin extract has characteristic peaks, which show the various vibrations of molecules and the interaction of resin extract with the second layer made of polypropylene. The interaction of resin extract with the second layer can be confirmed by the disappearance of O‐H stretching vibration in the extract‐coated second layer (Figure 5a).

The uncoated and coated third layer has the characteristic band at a range of 2950 to 719 cm^−1^. The uncoated and coated third layer shows a medium absorption band at 2950, 2916, and 2868 cm^−1^ due to the antisymmetric and symmetric stretching vibrations of the C─H bond in aliphatic compounds. The uncoated layer has medium absorption peaks at 2837 cm^−1^, and the coated layer has a peak at 2836 cm^−1^ indicating the stretching of the C─H bond of ‐CH_3_ attached to oxygen. The presence of a strong sharp peak at 1710 cm^−1^ in both coated and uncoated layers indicate the C═O stretching vibrations of α,β‐unsaturated aldehydes and ketones. The antisymmetric CH_3_ deformation in the uncoated layer is observed at 1451 cm^−1^, and in the coated layer, it is observed at 1453 cm^−1^. The in‐ring stretching of the C─C bond and in‐plane bending of OH present in carboxylic acid in the uncoated layer and coated layer can be observed at 1409 and 1405 cm^−1^ respectively. The medium absorption peak at 1370 cm^−1^ in both uncoated and coated layers indicated the rocking vibrational mode of the C‐H bond in alkanes. A strong peak at 1239 cm^−1^ in the coated and uncoated layer shows the stretching vibrations of the C─O─C bond in ether.^[^ ^34^ ^]^ A strong peak at 1089 cm^−1^ and a medium peak at 1015 cm^−1^ in the uncoated layer indicate the stretching vibrations of the C─O bond in alcohols and ethers. The strong band at 1092 cm^−1^ is observed on the coated layer due to the stretching mode of vibrations of the C─O bond in carboxylic acid.^[^ ^35^, ^36^, ^37^ ^]^ The ring breathing mode of carbon in cyclic compounds is noticed as a weak peak at 971 cm^−1^ in the uncoated layer and 969 cm^−1^ in the coated layer. The existence of a peak at 869 and 840 cm^−1^ in the uncoated layer and 868 and 841 cm^−1^ in the coated layer is due to the C─H bond showing out‐of‐plane bending vibration indicating the presence of an aromatic compound and O─O stretching in peroxides. The presence of an intense peak at 720 cm^−1^ indicates the out‐of‐plane deformation in phenols and the C─H bond in alkanes shows the rocking vibrational mode (Figure 5b).^[^ ^34^ ^]^

### 3.5 Wettability Analysis

**Figure 6:**
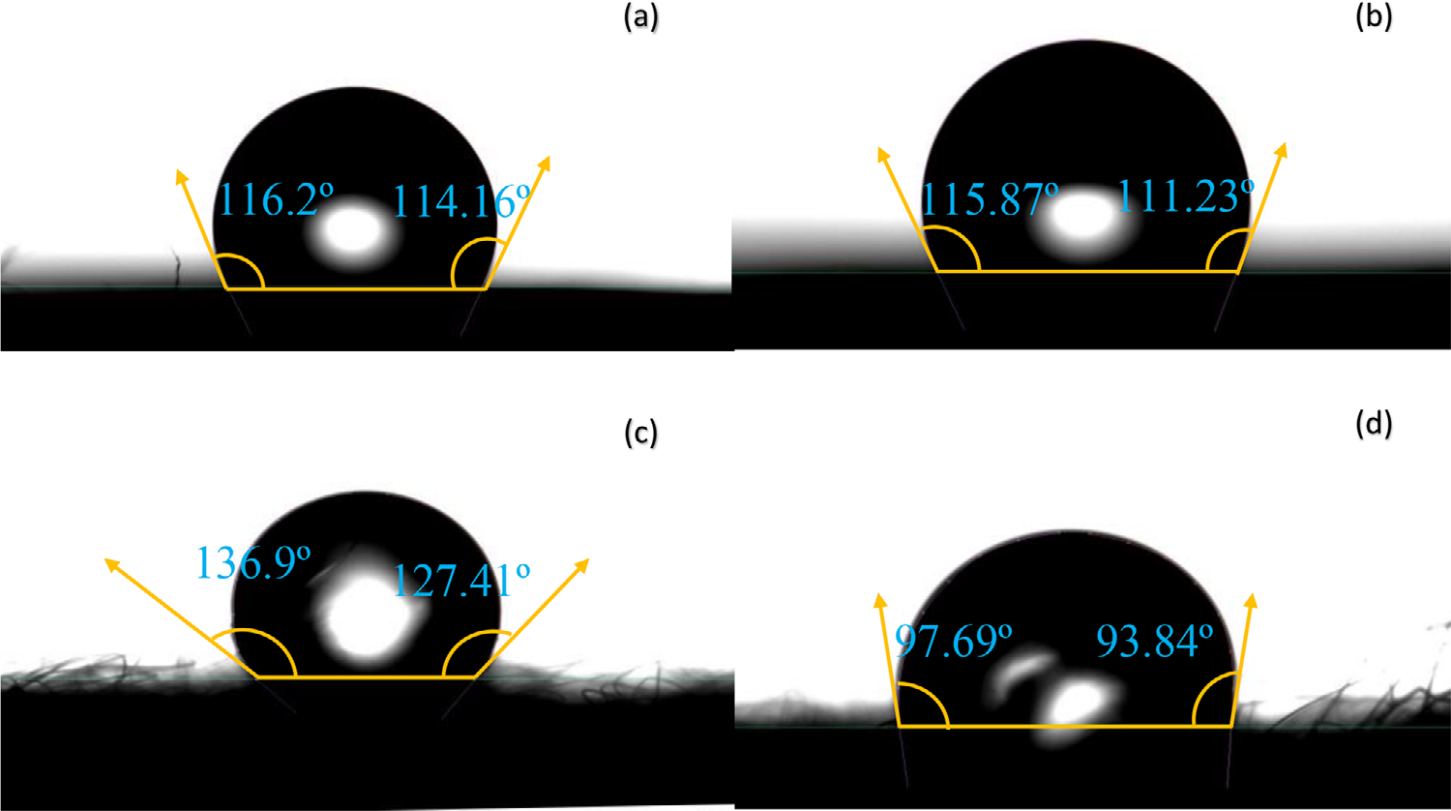
Water contact angle of a) second layer of mask, b) Boswellia resin extract‐coated second layer, c) third layer of mask, d) Boswellia resin extract‐coated third layer.

**Figure 7:**
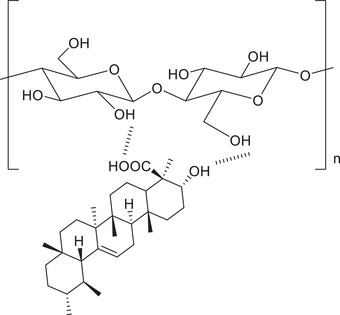
Interaction of the third layer of mask with boswellic acid.

The water contact angle analysis technique recorded the surface affinity of water on the surfaces of the material. It is evident that the water contact angle of materials with a hydrophobic nature is at an angle greater than 90°, while that of hydrophilic substances is less than 90°. By monitoring the water contact angle of the second and third layers of the mask coated with the BSE with the uncoated layers, we evaluated the surface affinity of the layers with water. The water contact angle of the uncoated second layer of the mask was observed as 116.2° on the left side and 114.16° on the right side of the surface (**Figure** **6**a). The second layer coated with Boswellia resin extract has a contact angle of 115.87° on the left side and 111.23° on the right side (Figure 6b). The coating with resin extract, does not result in any characteristic variation of the contact angle, and the hydrophobicity of the second layer is retained. The contact angle measured for the third layer was 136.9° on the left side and 127.41° on the right side (Figure 6c). The third layer coated with resin extract shows a contact angle of 97.69° and 93.84° on the left and right side respectively (Figure 6d). The water contact angle of the resin coated third layer is decreased, compared to the uncoated third layer. The decrease in the hydrophobicity of the coated layers of mask is due to the interaction of hydrophilic components present in the resin extract with the fabric of the mask. The main constituent in BSE is boswellic acid, which contains tunable carboxylic and hydroxyl functional groups. These functional groups can interact with the active hydroxyl group present in the third layer of the mask. The wettability of the Boswellia resin extract coated and uncoated second and third layer of the mask has been illustrated in (**Figure** **7**).^[^ ^38^, ^39^, ^40^, ^41^ ^]^

The contact angle measurements of the second and third layers after treating with BSE is due to the chemical interaction between the various functional group present in the extract with the mask layers. The main bioactive component present in the BSE is boswellic acid which has hydrophilic moieties due to the presence of functional groups such as ‐COOH and ‐OH groups. The second layer is made up of polypropylene and on interaction with extract, it retains the hydrophobic nature. While in the case of third layer, the contact angle is dropped from 136.9° to 97° because it is more receptive to interaction with hydrophilic component of the extract leading to the difference in the wettability of coated second and third layer.

### 3.6 Effect of *Boswellia Serrata* Extract on the Growth of Various Respiratory Pathogens Which Causes Air Borne Infections

**Figure 8:**
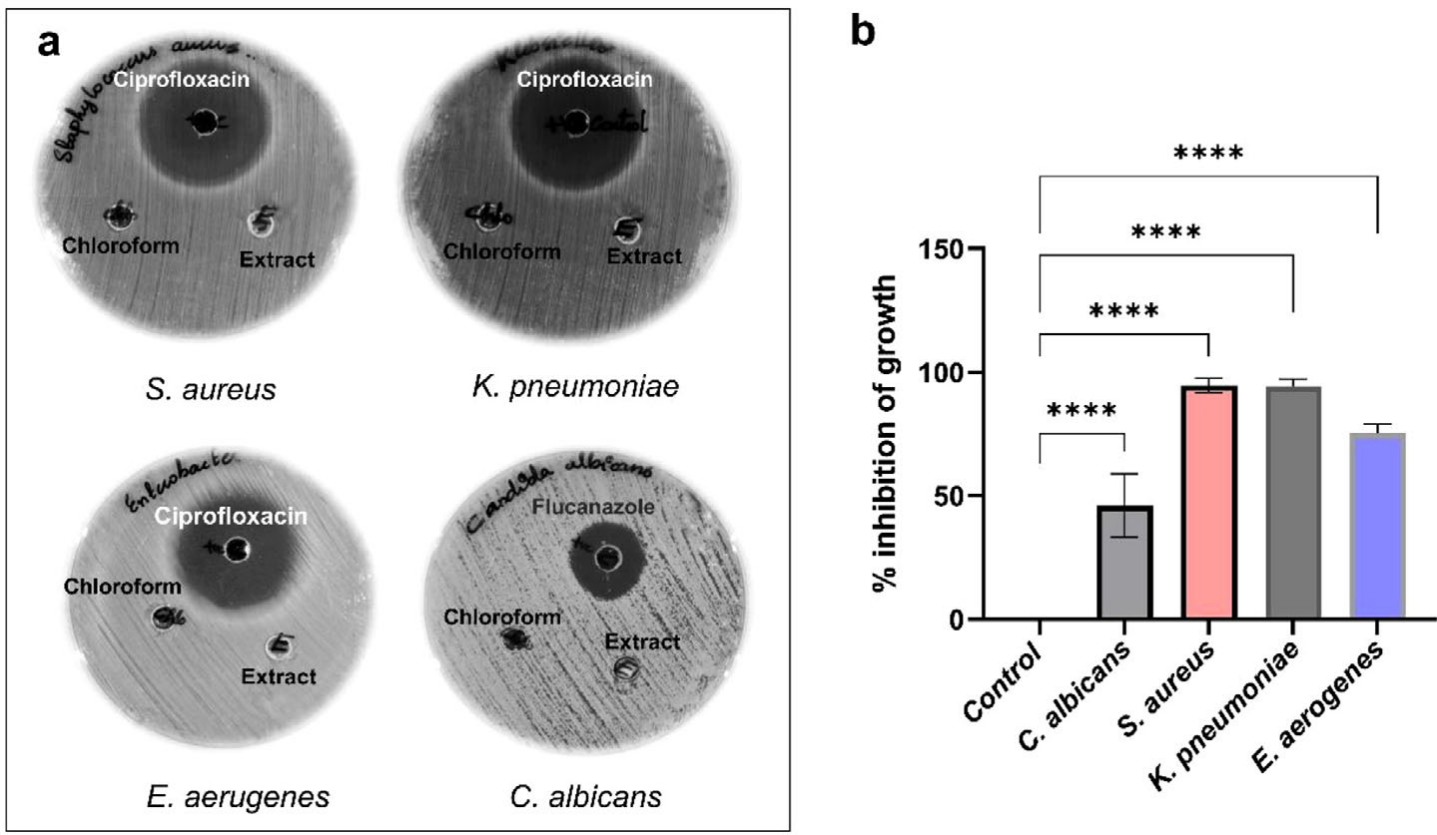
a) Effect of *Boswellia serrata* extract (100 µg mL^−1^) on the growth of different microorganisms by agar well diffusion method. b) Bars represent the percentage reduction of microbial growth compared to the untreated control grown in MH broth. Results are expressed as mean ± SD calculated from at least three independent experiments. One‐way ANOVA with multiple comparison test was performed **** represents *p* value = < 0.0001.

Initially, the anti‐microbial activity of BSE was investigated against three bacterial pathogens, which included *S. aureus, K. pneumoniae*, *E. aerogenes* and a fungal pathogen, *C. albicans*. Antimicrobial activity using agar well diffusion shows no significant zone of inhibition in any of these pathogens. This was suggestive of the poor diffusion or volatility of the extract in the agar. However, the broth dilution using 100 µg mL^−1^ of BSE demonstrated significant reduction in the growth of all the tested pathogens, when compared to the untreated control sample. Specifically, 94% and 75 % reduction in the growth of *K. pneumoniae, S. aureus* and 75% reduction in the growth *of E. aerogenes* was observed, as compared to the untreated control. In contrast, only 46% reduction was observed in the growth of *C. albicans* when treated with the BSE (**Figure** **8**). Ciprofloxacin was used as positive control and chloroform, which was used for dissolving the extract, was used as a solvent control. From the results, it is evident that the extract shows broad‐spectrum activity against and a wide range of mechanisms of action, including antibacterial and antifungal mechanisms.

These results are in agreement with the antimicrobial activity of twenty commercial frankincense essential oil samples as reported by Vuuren and coworkers.^[^ ^42^ ^]^ In addition to these reports, studies by^[^ ^43^ ^]^ confirmed the anti‐microbial activity of BSE, explicitly active against skin, scalp, and nail infections, triggered by microorganisms including *S. epidermidis* and *C. albicans*, clearly representing the potential of *Boswellia serrata* as a natural therapeutic agent for treating superficial infections. These results further validate the outcome of the present study and underscores the potential to use *Boswellia serrata* as a promising anti‐microbial agent for various applications.

### 3.7 Antimicrobial Potency of *Boswellia Serrata* Extract‐Coated Mask Layers

**Figure 9:**
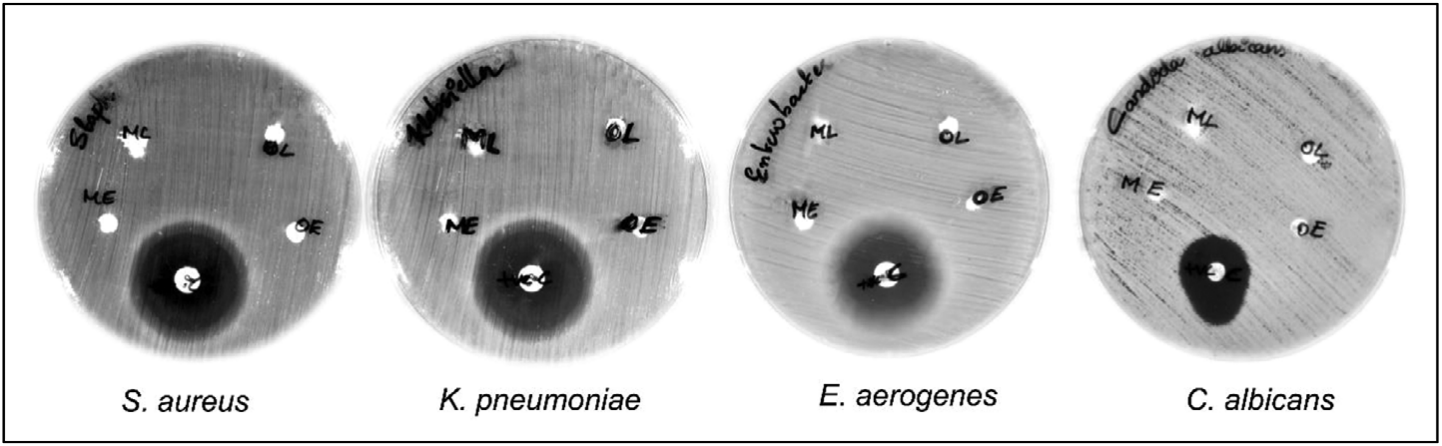
Antimicrobial activity of *Boswella serrata* extract coated on the second and third layer of N95 mask material by disc diffusion method. Ciprofloxacin was used as a positive control for *S. aureus*, *K. pneumoniae*, and *E. aerogenes*, while Fluconazole was used as a positive control for *C. albicans*.

As the BSE unequivocally revealed profound antibacterial activity against various pathogens, the second and third layers of the N95 mask were integrated with BSE and the functionalized mask layer fabrics were then tested for their efficacy in reducing the microbial load. The antibacterial and antifungal activities of BSE infused onto a polypropylene (second layer) and cotton filter (third layer) of N95 mask material, were investigated against various pathogens, specifically those implicated in airborne infections. The bacterial species used in this study, included the gram‐negative *E. aerogenes*,^[^ ^44^ ^]^ and *K. pneumoniae*,^[^ ^45^, ^46^ ^]^ the gram‐positive *S. aureus* and the fungi *Candida* ^[^ ^47^ ^]^ have been implicated in airborne infections. Contact time‐kill assay was used to determine the microbicidal activity of BSE coated on the polypropylene (second) layer and cotton (third) layer of N95 mask material. There was no significant inhibition of the growth of microorganisms in coated and uncoated mask materials as elucidated by agar diffusion assays (**Figure** **9**). The absence of inhibition of growth of microorganism in diffusion assay emphasizes the combined hydrophobic nature of the BSE incorporated into the matrix materials. However, the spread plate assay to check the contact killing of microbes at different time points, demonstrated a significant reduction in the microbial load after 1, 4 and 8 h of incubation. The graphs (**Figure** **10**) indicate the corresponding percentage inhibition of *E. aerogenes, K. pneumoniae, S. aureus*, and *C. albicans* in the mask materials. The extract coated on the second layer of the mask material shows 19.44 % reduction in the growth of fungal pathogen *C. albicans* after 1 h. However, there was no reduction in *C. albicans* growth in the third layer. However, the second and third layers displayed strong anti‐microbial activity at 4 h of 81.4% and 100% respectively. The anti‐microbial activity of both layers against *C. albicans* further decreased drastically after 8 h of contact, reaching 61 % and 75 % for the second and third layers, respectively (Figure 10a).

Figure 10Colony forming units of *C. albicans*, *E. aerogenes*, *K. pneumoniae*, and *S. aureus* formed after contact killing assay of the second and third layer of mask material in uncoated and *Boswellia serrata* extract‐coated layers of mask material at three different time intervals: a) 1 h, b) 4 h and c) 8 h. d) Percentage reduction of microbial load in extract‐coated and uncoated second layer and third layer of the mask material at different time intervals (1 and 8 h). Results are expressed as mean ± SD calculated from at least three independent experiments. 2‐way ANOVA with multiple comparison test was performed ****p < 0.0001; ***p < 0.001; **p < 0.01; *p < 0.1; ns‐not significant.

**Figure:**
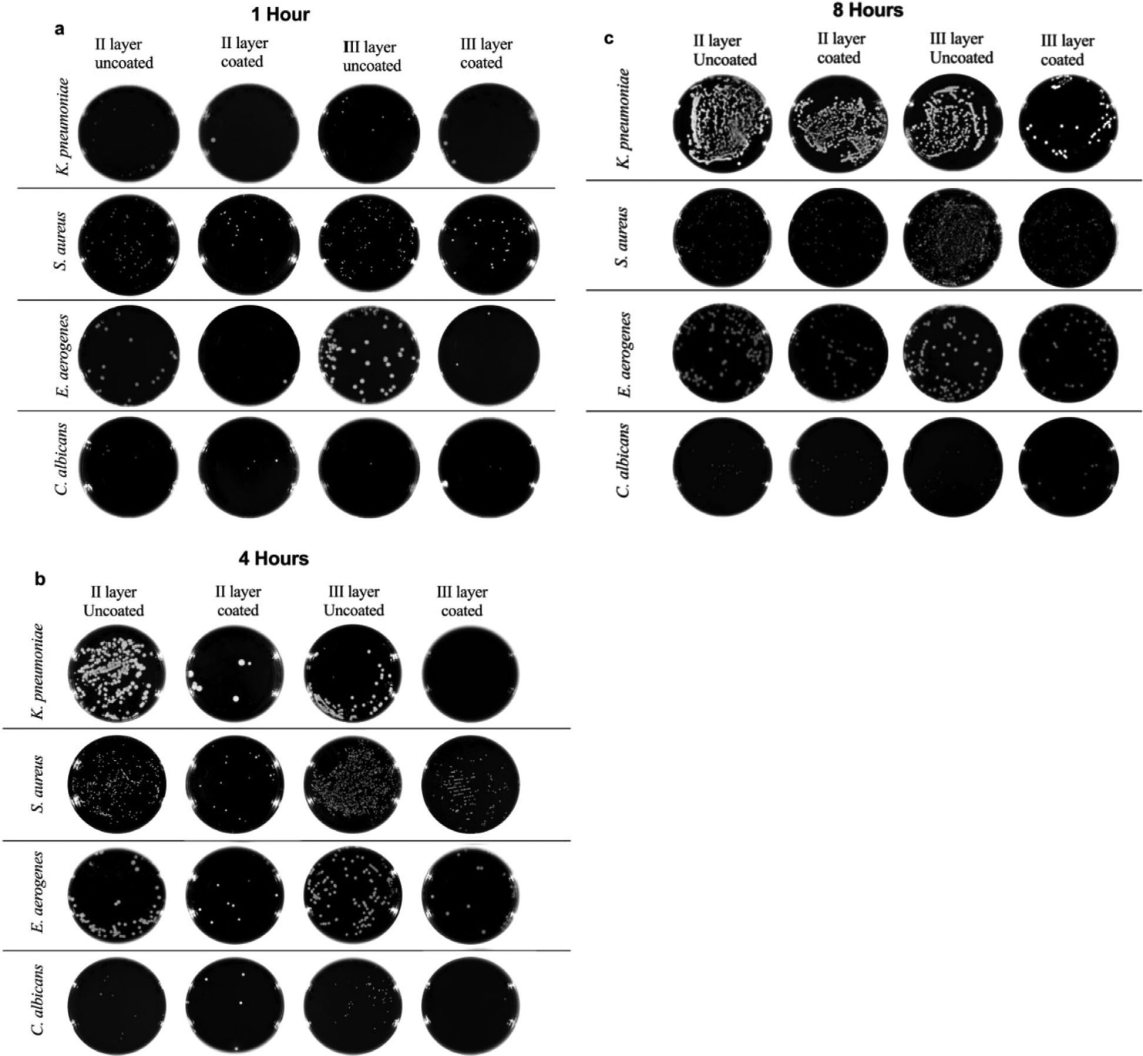

**Figure:**
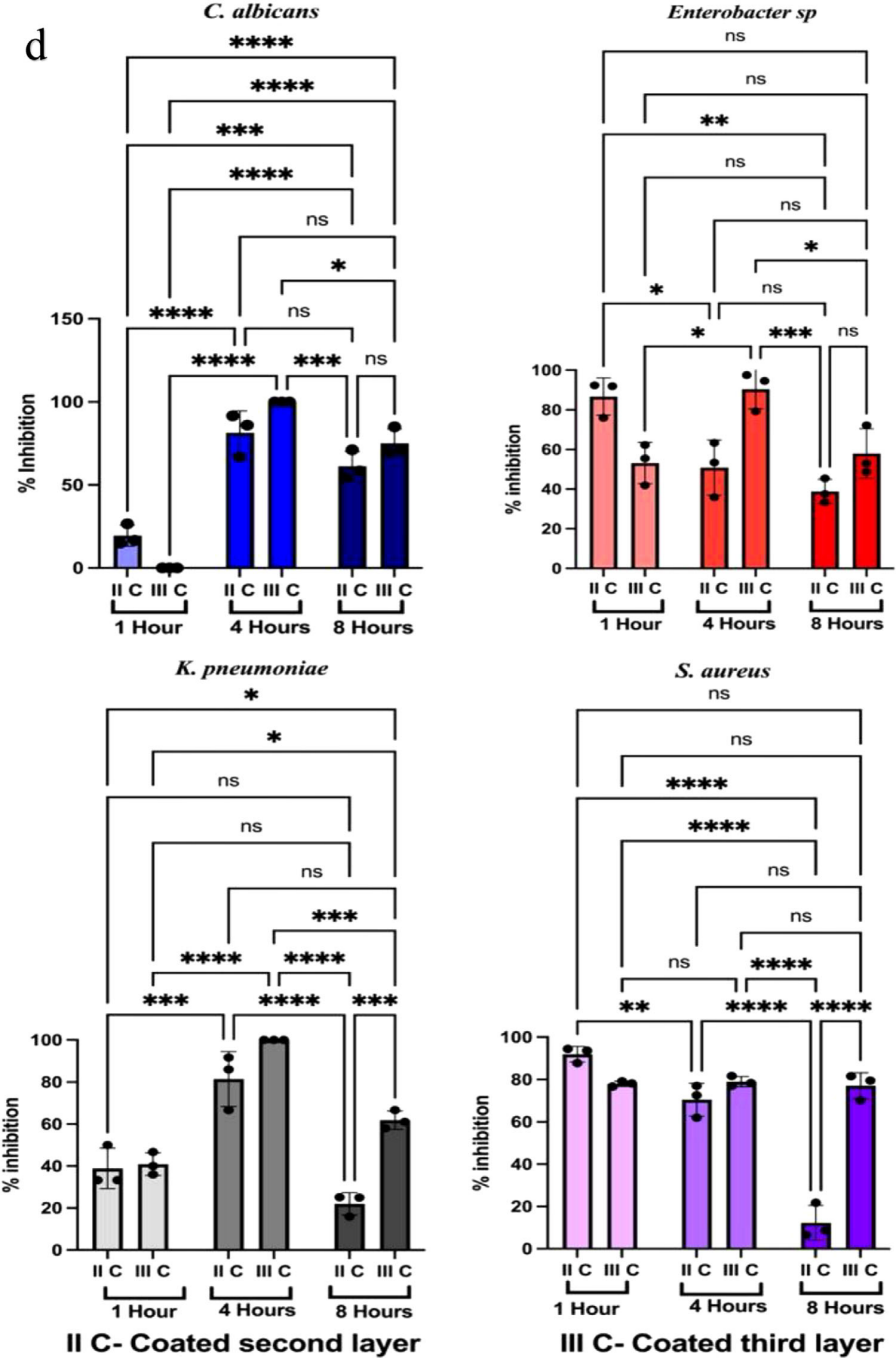

The second layer showed an initial inhibition of 86.7% of growth of *E. aerogenes* after 1 h of contact with extract coated mask material pieces. However, an extended duration of contact for 4 and 8 h reduced the anti‐bacterial activity, achieving only up to 50.9% and 38.7% reduction in microbial growth. However in third layer at 1 h of incubation there was 53.2% reduction in the growth of *E. aerogenes*, conversely by 4 h, the antibacterial activity was enhanced by 90.4%. But by 8th h it was again reduced to 57.9% The extract‐coated second and third layers of the mask material demonstrated antibacterial activity against *K. pneumoniae*. After 1 h of contact, the second layer showed 38.8% and the third layer exhibited 40.8% inhibition of bacterial growth. However, by 4th h the antibacterial efficacy was enhanced by 81.4%. After prolonged contact for 8 h, the inhibitory effect of the second layer was substantially decreased to 24.1%, whereas the third layer exhibited a significant increase in activity, from 1 h to 4th h reaching 40.8% to 100% and reducing to 61.9% inhibition by 8th h. (Figure 10b).

The contact‐killing activity of the second and third layers of the coated mask material against *S. aureus* for one hour was found to be 92.4% and 78.0 %. The antibacterial activity of II layer reduced to 70.5% by 4 h and further reduced to 12.6 % by 8th h. Further, after 4 h, the activity of the third layer, increased to 79% (Figure 10b). The growth inhibition of second and third layers on various pathogens at different time intervals (1, 4 and 8 h) is tabulated in **Table** **2**. The different time‐dependent antimicrobial efficacy of the extract‐coated mask material against the common respiratory pathogen indicates its safe usage to reduce the infection rate.

**Table 2:** Percentage inhibition of the growth of microbes at *Boswellia serrata* extract coated second and third layer at different time points.

Organism	% Inhibition of growth					
	Second layer			Third layer		
	1 h	4 h	8 h	1 h	4 h	8 h
*C. albicans*	19.44	81.4	61.36	0	100	75.09
*S. aureus*	92.46	70.50	12.65	78.03	79	77.23
*K. pneumoniae*	38.88	81.4	24.13	40.88	100	61.92
*E. aerogenes*	86.74	50.9	38.76	53.22	90.4	57.94

The antimicrobial effect observed in the second layer of the mask material showed a rapid decline within 1–8 h, which may be attributed to the weak interaction of hydrophobic and hydrophilic group in the material and extract respectively. The second layer is made up of polypropylene which is inherently hydrophobic in nature. This non‐polar nature of material have weak affinity toward the polar groups carrying components in the resin extract such as boswellic acid. So that, it does not strongly adhere to the polypropylene layer rather than a temporary interaction leads to reduced retention and faster release of the coated extract, thereby limiting its sustained activity. In contrast, the third layer is made up of demonstrated a more stable and prolonged antimicrobial response, maintaining a higher percentage of inhibition against the tested pathogens. This suggests that the third layer plays a crucial role in preserving the overall antimicrobial efficacy of the mask material.

These findings imply that the third layer coated with BSE exhibited more sustained and long‐lasting antimicrobial activity. While the second layer showed rapid and short‐term antimicrobial effect which may be attributed to the reduced retention and faster release of the coated extract, thereby limiting its sustained activity. Hence, coating the third layer of the mask material with BSE provides a superior and consistent microbial protection, making it the better choice for long‐term anti‐microbial activity.

### 3.8 Bacterial Filtration Efficiency and Breathability

Compared to the second layer of coated mask material, the third layer shows significant activity. So, we analyzed bacterial filtration efficiency (BFE) IS 16 288 (**Table** **3**). It shows an efficiency of 99.2%. This test passed in Class *1, 2 and 3* of the specified standard.

**Table 3:** Bacterial filtration efficiency of third layer.

Average plate of positive control	2292
Average plate of negative control	0
Bacterial filtration efficiency (%)	99.2

**Figure 11:**
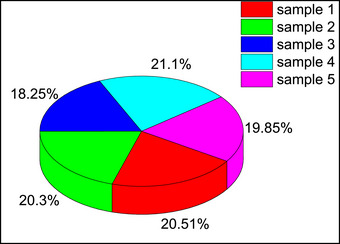
Differential pressure of the third layer of the mask material.

The breathability of the third layer of mask fabric is analyzed using five samples (sample 1, 2 3 4 and 5). It is found that the differential pressure of the functionalized layer ranges from 21.89 to 25.31 Pa, which has an average pressure drop of 23.99 Pa. Hence, the functionalized layer meets requirements specified in IS 16 289. This test result passed in Class *1, 2 and 3* of the specified standard (**Figure** **11**).

## 4 Conclusion

This study established the efficacy of BSE as an environmentally friendly, bioactive agent for imparting antibacterial as well as antifungal properties to both cotton and polypropylene substrates. Utilizing a dip‐coating technique, the extract was successfully incorporated into the second and third layers of the N95 mask.

Specifically, the third layer of the mask, when coated with BSE, demonstrated significant antimicrobial activity against *C. albicans* and S. *aureus* within 8 h of contact, while the second layer exhibited activity against *K. pneumoniae* and *E. aerogenes* within the first hour. FTIR spectroscopy confirmed the successful integration of the active constituents of the BSE, onto both the second and third mask layers, while contact angle measurements revealed the enhanced hydrophobic nature of the coated surfaces.

The present study substantiated that the active components within the BSE validated to be an efficacious bactericidal agent against both Gram‐negative and Gram‐positive bacteria as well as an antifungal agent by inhibiting colony formation. The dip‐coating method is applied for the BSE on mask layers highlights the potent antimicrobial capabilities of the coated material. Such a advancement holds significant promise for fabricating commercial masks with inherent disinfection properties, particularly leveraging the enhanced hydrophobicity, to effectively prevent airborne disease‐causing agents.

## Conflict of Interest

The authors declare no conflict of interest.

## Data Availability

The data that support the findings of this study are available from the corresponding author upon reasonable request.

## References

[1] A. Tcharkhtchi , N. Abbasnezhad , M. Zarbini Seydani , N. Zirak , S. Farzaneh , M. Shirinbayan , Bioact. Mater. 2021, 6, 106. 32817918 10.1016/j.bioactmat.2020.08.002 PMC7426537

[2] R. Macintyre , A. A. Chughtai , Bmj. 2015, 350. 10.1136/bmj.h694 25858901

[3] C. R. MacIntyre , A. A. Chughtai , B. Rahman , Y. Peng , Y. Zhang , H. Seale , X. Wang , Q. Wang , Influenza Other Respir. Viruses 2017, 11, 511. 28799710 10.1111/irv.12474 PMC5705692

[4] C. B. Hiragond , A. S. Kshirsagar , V. V. Dhapte , T. Khanna , P. Joshi , P. V. More , Vacuum 2018, 156, 475.

[5] M. Hashmi , S. Ullah , I. S. Kim , Curr. Res. Biotechnol. 2019, 1, 1.

[6] V. Gopal , B. E. Nilsson‐Payant , H. French , J. Y. Siegers , B. R. tenOever , W.‐S. Yung , M. Hardwick , A. J. W. Te Velthuis , bioRxiv 2021, 11, 2020. 10.1021/acsami.1c04412 PMC8262172 34180223

[7] E. Horváth , L. Rossi , C. Mercier , C. Lehmann , A. Sienkiewicz , L. Forró , Adv. Funct. Mater. 2020, 30, 2004615. 32837497 10.1002/adfm.202004615 PMC7435547

[8] F. A. Monge , P. Jagadesan , V. Bondu , P. L. Donabedian , L. Ista , E. Y. Chi , K. S. Schanze , D. G. Whitten , A. M. Kell , ACS Appl. Mater. Interfaces 2020, 12, 55688. 33267577 10.1021/acsami.0c17445 PMC7724758

[9] H. Zhong , Z. Zhu , P. You , J. Lin , C. F. Cheung , V. L. Lu , F. Yan , C. Y. Chan , G. Li , ACS Nano 2020, 14, 8846. 32578981 10.1021/acsnano.0c03504

[10] X. Shan , H. Zhang , C. Liu , L. Yu , Y. Di , X. Zhang , L. Dong , Z. Gan , ACS Appl. Mater. Interfaces 2020, 12, 56579. 33259195 10.1021/acsami.0c16754

[11] M. Carugno , F. Dentali , G. Mathieu , A. Fontanella , J. Mariani , L. Bordini , G. P. Milani , D. Consonni , M. Bonzini , V. Bollati , A. C. Pesatori , Environ. Res. 2018, 166, 452. 29940478 10.1016/j.envres.2018.06.016

[12] G. Chen , W. Zhang , S. Li , G. Williams , C. Liu , G. G. Morgan , J. J. K. Jaakkola , Y. Guo , Environ. Res. 2017, 156, 306. 28388516 10.1016/j.envres.2017.03.046

[13] N. Chen , J. Shi , J. Huang , W. Yu , R. Liu , L. Gu , R. Yang , Z. Yu , Q. Liu , Y. Yang , S. Cui , Z. Wang , BMC Public Health 2020, 20, 447. 32252726 10.1186/s12889-020-8423-4 PMC7132958

[14] J. Rylance , A. Kankwatira , D. E. Nelson , E. Toh , R. B. Day , H. Lin , X. Gao , Q. Dong , E. Sodergren , G. M. Weinstock , R. S. Heyderman , H. L. Twigg , S. B. Gordon , BMC Microbiol. 2016, 16, 182. 27514621 10.1186/s12866-016-0803-7 PMC4982214

[15] J. M. Sahuquillo‐Arce , E. Ibáñez‐Martínez , A. Hernández‐Cabezas , A. Ruiz‐Gaitán , P. Falomir‐Salcedo , R. Menéndez , J. L. López‐Hontangas , ERJ Open Res. 2017, 3, 00014. 29209621 10.1183/23120541.00014-2017 PMC5709705

[16] D. de M. Castro e Silva , R. M. N. Marcusso , C. G. G. Barbosa , F. L. T. Gonçalves , M. R. A. Cardoso , Heliyon 2020, 6, 05065. 10.1016/j.heliyon.2020.e05065 PMC7550922 33083593

[17] D. R. Kollath , J. R. Mihaljevic , B. M. Barker , Microbiol. Spectr. 2022, 10, 0148321. 10.1128/spectrum.01483-21 PMC9045372 35319247

[18] P. Y. Liu , Y. T. Tsan , Y. W. Chan , W. C. Chan , Z. Y. Shi , C. T. Yang , B. S. Lou , J. Ambient. Intell. Humaniz. Comput. 2024, 15, 1837.

[19] Y. Vithal Patil , N. R. Pagare , S. M. Wakchoure , O. A. Patil , P. H. Pawar , Y. Patil , Int. J. Res. Edu. Sci. Methods 2024, 12, 00014.

[20] R. H. Caliebe , T. Scior , H. P. T. Ammon , Arch. Pharm. 2021, 354, 2100160. 10.1002/ardp.202100160 PMC8646807 34427335

[21] A. A. Gomaa , H. S. Mohamed , R. B. Abd‐ellatief , M. A. Gomaa , Inflammopharmacology 2021, 29, 1033. 34224069 10.1007/s10787-021-00841-8 PMC8256410

[22] R. Chandran Rema , A. Salim , R. Babu , S. Pal , R. Biswas , B. N. Sathy , S. V. Nair , D. Menon , ACS Appl. Polym. Mater 2022, 4, 4839.

[23] P. Nisha , R. M. S. , S. Devanesan , T. A. Mir , R. Chinnappan , M. S. AlSalhi , T. Alzahim , Environ. Technol. Innov. 2024, 36, 103764.

[24] S. Niveditha , V. T. Veetil , A. D. Rajeeve , S. Cheriyan , R. Yamuna , M. Karthega , J. Drug Deliv. Sci. Technol. 2024, 95, 105597.

[25] P. Haripriya , M. P. Revathy , M. S. Kumar , P. Navaneeth , P. V. Suneesh , T. G. Satheesh Babu , V. R. K. Darbha , Nanotechnology 2024, 35, ad1d15. 10.1088/1361-6528/ad1d15 38198713

[26] B. S. Haripriya , D. R. Anakha , R. Yamuna , M. Vinoba , M. Bhagiyalakshmi , J. Porous Mater. 2024, 31, 351.

[27] S. Thirumal , G. Duraikannu , Int. J. Pharm. Biol. Sci. Arch. 2019.

[28] D. Bigman , H. Fofack , Eur. Biophys. J. 2001, 30, 250.11548127

[29] J. Hayat , M. Akodad , A. Moumen , M. Baghour , A. Skalli , S. Ezrari , S. Belmalha , Heliyon 2020, 6, 05609. 10.1016/j.heliyon.2020.e05609 PMC7708819 33305038

[30] C. Guo , H. Liu , J. Wang , J. Chen , J. Colloid Interface Sci. 1999, 209, 368. 9885264 10.1006/jcis.1998.5897

[31] X. Liu , C. M. G. C. Renard , S. Bureau , C. Bourvellec , Carbohydr. Polym. 2021, 262, 117935. 33838812 10.1016/j.carbpol.2021.117935

[32] R. M. Khafagy , Y. A. Badr , J. Polym. Sci. B Polym. Phys. 2005, 43, 2829.

[33] S. A. Hedrick , S. S. C. Chuang , Thermochim. Acta 1998, 315, 159.

[34] H. F. Shurvell , in Handbook of Vibrational Spectroscopy, 3, 2006 pp. 1783–1816.

[35] T. N. T. Rohadi , M. J. M. Ridzuan , M. S. A. Majid , E. M. Cheng , M. J. Norasni , N. Marsi , J. Phys. Conf. Ser. 2021, 2051, 012024.

[36] S. Kumar , A. Mudai , B. Roy , I. B. Basumatary , A. Mukherjee , J. Dutta , Foods 2020, 9, 1143. 32825205 10.3390/foods9091143 PMC7555077

[37] R. L. Frost , Y. Xi , Spectrochim. Acta A Mol. Biomol. Spectrosc. 2013, 103, 151. 23257343 10.1016/j.saa.2012.11.031

[38] G. S. Selvam , T. Dheivasigamani , A. Prabhu , N. K. Mani , ACS Omega 2022, 7, 24606. 35874217 10.1021/acsomega.2c02337 PMC9301725

[39] S. Sudarsan , P. Shetty , R. Chinnappan , N. K. Mani , Anal. Bioanal. Chem. 2023, 415, 6449. 37665340 10.1007/s00216-023-04921-2 PMC10567893

[40] A. Marmur , C. Della Volpe , S. Siboni , A. Amirfazli , J. W. Drelich , Surf. Innov. 2017, 5, 3.

[41] S. Sudarsan , P. Shetty , R. Chinnappan , N. K. Mani , Anal. Bioanal. Chem. 2023, 415, 6449. 37665340 10.1007/s00216-023-04921-2 PMC10567893

[42] S. F. Van Vuuren , G. P. P. Kamatou , A. M. Viljoen , S. Afr. J. Bot. 2010, 76, 686.

[43] S. Sadhasivam , S. Palanivel , S. Ghosh , Lett. Appl. Microbiol. 2016, 63, 495. 27730658 10.1111/lam.12683

[44] D. I. Clemmer , J. L. S. Hickey , J. F. Bridges , D. J. Schliessmann , M. F. Shaffer , J. Infect. Dis. 1960, 106, 197. 13810652 10.1093/infdis/106.2.197

[45] R. A. Fodah , J. B. Scott , H. H. Tam , P. Yan , T. L. Pfeffer , R. Bundschuh , J. M. Warawa , PLoS One 2014, 9, 107394,. 10.1371/journal.pone.0107394 PMC4159340 25203254

[46] K. Skowron , N. Wiktorczyk , J. Kwiecińska‐Piróg , A. Sękowska , E. Wałecka‐Zacharska , E. Gospodarek‐Komkowska , Lett. Appl. Microbiol. 2019, 69, 333. 31536642 10.1111/lam.13223

[47] E. M. Rick , K. Woolnough , C. H. Pashley , A. J. Wardlaw , J. Investig. Allergol. Clin. Immunol. 2016, 26, 344. 10.18176/jiaci.0122 27996940

